# Physical activity and sedentary time in relation to academic achievement in children

**DOI:** 10.1016/j.jsams.2016.11.003

**Published:** 2017-06

**Authors:** Eero A. Haapala, Juuso Väistö, Niina Lintu, Kate Westgate, Ulf Ekelund, Anna-Maija Poikkeus, Soren Brage, Timo A. Lakka

**Affiliations:** a Institute of Biomedicine/Physiology, University of Eastern Finland, Kuopio Campus, Finland; b Childhood Health and Active Living Research Group, Department of Biology of Physical Activity, University of Jyväskylä, Finland; c Institute of Dentistry, University of Eastern Finland, Kuopio Campus, Finland; d MRC Epidemiology Unit, University of Cambridge, University of Cambridge School of Clinical Medicine, Institute of Metabolic Science, Cambridge Biomedical Campus, United Kingdom; e Department of Sport Medicine, Norwegian School of Sports Science, Norway; f Department of Teacher Education, University of Jyväskylä, Finland; g Department of Clinical Physiology and Nuclear Medicine, University of Eastern Finland, Kuopio Campus, Finland; h Kuopio Research Institute of Exercise Medicine, Finland

**Keywords:** Motor activity, Sedentary behavior, Academic performance, Cognition, Pediatrics

## Abstract

**Objectives:**

To investigate the independent and combined associations of objectively measured moderate-to-vigorous physical activity (MVPA) and sedentary time (ST) with reading and arithmetic skills.

**Design:**

Cross-sectional/prospective.

**Methods:**

Participants were 89 boys and 69 girls aged 6–8 years. MVPA and ST were measured using a combined heart rate and movement sensor and body fat percentage by dual-energy X-ray absorptiometry in Grade 1. Reading fluency, reading comprehension, and arithmetic skills were assessed using standardized tests in Grades 1–3. The data were analyzed using linear regression analyses and analyses of covariance with repeated measures.

**Results:**

In boys, MVPA was directly and ST inversely associated with reading fluency in Grades 1–3 and arithmetic skills in Grade 1 (*P* < 0.05). Higher levels of MVPA were also related to better reading comprehension in Grade 1 (*P* < 0.05). Most of the associations of MVPA and ST with reading and arithmetic skills attenuated after mutual adjustment for MVPA or ST. Furthermore, boys with a combination of lower levels of MVPA and higher levels of ST had consistently poorer reading fluency (*P* = 0.002) and reading comprehension (*P* = 0.027) across Grades 1–3 than other boys. In girls, ST was directly associated with arithmetic skills in Grade 2 (*P* < 0.05). However, this relationship of ST with arithmetic skills was no longer significant after adjustment for body fat percentage.

**Conclusions:**

Lower levels of MVPA and higher levels of ST and particularly their combination were related to poorer reading skills in boys. In girls, higher levels of ST were related to better arithmetic skills.

## 1 Introduction

Physical inactivity is a major public health problem in developed countries.^1^ There is some evidence that higher levels of physical activity (PA) and lower levels of sedentary time (ST) are associated with better brain structures and functions in children.[2], [3] Moreover, low levels of PA and high levels of ST, particularly TV watching, have been linked to poorer academic achievement in children.[4], [5] However, these relationships are mainly based on data from cross-sectional studies using self-reported PA and ST.[4], [6] The ability of self-report to rank the level of PA correctly has been questioned, and self-reports are also prone to recall bias.^7^ Therefore, evidence on the associations of objectively measured PA and ST with academic achievement in children is highly warranted.

Higher levels of objectively measured moderate-to-vigorous PA (MVPA) at the age of 11 were associated with better academic achievement at the ages of 11, 13, and 16 years^8^ but with poorer academic achievement in children and adolescents aged 6–18 years.^9^ Some studies have found no relationship between objectively measured MVPA and academic achievement in children 10 years of age.[5], [10] In recent studies, higher levels of PA have been associated with better academic achievement only among boys.[11], [12] In contrast, some studies have observed an association between higher levels of PA and better cognitive performance in girls but not in boys.[13], [14] There are studies on the associations of MVPA with academic achievement during childhood, although MVPA accounts only for a small proportion of daily PA among children.^15^ However, there are no studies on the relationships of objectively measured light PA to academic achievement although children spent most of their physically active time in light PA.^15^

Few available studies on the associations of objectively measured ST with academic achievement suggest a weak positive or no relationship between ST and academic achievement in children^5^ and adolescents.[16], [17] These results are consistent with the observations indicating that some sedentary behaviors, such as TV watching, are inversely associated whereas some sedentary behaviors, such as reading, are directly associated with academic achievement in children that makes the interpretation of the associations between total ST and academic achievement difficult.^4^

The evidence on the independent and combined relationships of objectively measured PA and ST to academic achievement is limited. Furthermore, there are only a few studies on the longitudinal associations of PA and ST with academic achievement during the first school years. Therefore, we investigated the independent and combined associations of objectively measured MVPA, light PA, and ST in Grade 1 with reading and arithmetic skills in Grades 1–3 in Finnish boys and girls aged 6–8 years.

## 2 Methods

### 2.1 Study design and study population

Data for the present analyses were obtained from the Physical Activity and Nutrition in Children (PANIC) Study and the First Steps Study, two independent studies that are being conducted simultaneously among primary school children in the City of Kuopio, Finland.^18^ Altogether 207 children from the City of Kuopio participated in both the PANIC Study and the First Steps Study. Data on PA, ST, and confounding factors were derived from the PANIC Study in Grade 1 and data on reading and arithmetic skills at the end of Grades 1–3 were received from the First Steps Study. Complete data on variables used in the present analyses were available for 153 children (89 boys, 64 girls) in Grade 1, 149 children (87 boys, 62 girls) in Grade 2, and 145 children (86 boys, 59 girls) in Grade 3. Children who were excluded from the present analyses because of incomplete data had higher levels of MVPA, lower levels of ST, and higher maximal workload per lean body mass in maximal cycle ergometer exercise test than the children who were included (P < 0.05). There were no differences in other characteristics between children in the study sample and the excluded children. The PANIC Study protocol was approved by the Research Ethics Committee of the Hospital District of Northern Savo, Kuopio, and the First Steps Study protocol was approved by the Research Ethics Committee of the University of Jyväskylä. All participating children and their parents provided written informed consent. The funding sources had no role in the collection, analysis, or interpretation of the data or in the approval or disapproval of the publication.

### 2.2 Assessment of academic achievement

Reading fluency was assessed using a group-administered subtest of the nationally normed reading achievement test battery for primary schools called Ala-asteen lukutesti (ALLU) in Finnish.^19^ The test score was the number of correct answers, ranging from 0 to 80, during a 2-min time limit for items that involved identifying the correct word from four phonologically similar alternatives linked to an adjoining picture.

Reading comprehension was assessed with a group-administered subtest from the ALLU test battery.^19^ After reading a short text, children were asked to answer to 12 multiple-choice questions relating to facts, causal relationships, interpretations, or conclusions drawn from the text. The test score was the number of correct answers, ranging from 0 to 12, during the 30-min test period when children were allowed to refer to the original text.

Arithmetic skills were assessed using a basic arithmetic test with a set of visually presented addition and subtraction tasks.^20^ Children were asked to perform as many calculations as they could during the 3-min time limit. The test score was the number of correct answers, ranging from 0 to 28.

### 2.3 Assessment of physical activity and sedentary time

PA was objectively assessed using a combined heart rate and movement sensor (Actiheart^®^, CamNtech Ltd., Papworth, UK)^21^ which was attached to the children’s chest with two standard ECG electrodes. The children were asked to wear the sensor continuously for a minimum of four days (including sleep and water-based activities) without changing their usual behavior. The heart rate data were individually calibrated with data from a maximal cycle ergometer exercise test. We defined MVPA as activities exceeding the intensity of 4 metabolic equivalents (METs), light PA as activities that were performed at the intensity of 1.5–4 metabolic equivalents (METs), and ST as activities that were performed below the intensity of 1.5 METs. We included data on children who had at least 48 h (32 h during weekdays, 16 h during weekend days, represented by ≥12 h of morning, noon, afternoon, and evening wear time) of valid activity recording in the analyses.^22^

### 2.4 Other assessments

Body height and body weight were measured by standard procedures.^23^ Pubertal status was assessed using the five stage criteria described by Tanner.^24^ Body fat mass, body fat percentage, and lean body mass were measured using the Lunar Prodigy Advance^®^ DXA device.^25^ Cardiovascular fitness (maximal workload per lean body mass) was assessed by a maximal cycle ergometer test and motor performance by 50-m shuttle run test time.^25^ Parental education, household income, PA at different settings including unsupervised PA, organized sports, other supervised exercise, PA at recess, and physically active school transportation, habitual time spent watching TV, using computer, and reading were assessed by a questionnaire.^26^ The risk of reading disability was assessed in the First Steps Study as described earlier.^27^ Children’s wellbeing was assessed by a questionnaire that was filled out by the parents and included 37 questions on the frequency of the components of physical, psychological, and social wellbeing scored between 1 (never) and 5 (every day or almost every day). Overall wellbeing score was computed as a sum of all these measures that ranged between 37 and 185, lower score indicating better overall wellbeing.

### 2.5 Statistical methods

We performed all data analyses using SPSS Statistics, Version 21.0 (IBM Corp., Armonk, NY, USA). Basic characteristics between boys and girls were compared using the Student’s t-test, the Mann–Whitney U-test, or the chi square-test. The associations of MVPA, light PA, and ST in Grade 1 with reading fluency, reading comprehension, and arithmetic skills in Grades 1–3 were studied using linear regression analyses. MVPA, Light PA, and ST were entered into the model one by one and data were adjusted for age, sex, and sensor wear time. The data were mutually adjusted for MVPA, light PA, and ST in order to investigate the independent associations of these measures with academic achievement. We additionally adjusted the data on the associations of PA and ST with academic skills in Grades 2–3 for academic skills in Grade 1.

The combined associations of MVPA and ST in Grade 1 with academic achievement in Grades 1–3 were investigated by dichotomizing MVPA and ST at their sex-specific medians and comparing academic achievement of children with a combination of lower levels of MVPA (≤109 min/d for boys, ≤79 min/d for girls) along with higher levels of ST (>215 min/d for boys, >227 min/d for girls) to all other children using analyses of covariance with repeated measures adjusted for age, sex, and sensor wear time. We investigated the interactions of sex with PA and ST on academic skills using general linear models.

In addition to the adjustment for age, sex, and sensor wear time, all data were further adjusted for parental education, household income, maximal workload achieved in exercise test, 50-m shuttle run test time, body fat percentage, PA at different settings, time spent watching TV, using a computer, reading or writing, risk for reading disability, or the wellbeing score.

## 3 Results

Boys were slightly older, had a lower body fat percentage, a higher maximal workload achieved in exercise test, and a faster 50-m shuttle run test time, and were more likely to have increased risk for reading disability than girls (Table 1). Boys also had higher levels of MVPA, poorer reading comprehension in Grade 2, and poorer reading fluency in Grade 3 than girls.

In all children, MVPA in Grade 1 was directly associated with reading fluency in Grades 2 and 3 (Table 2). MVPA or ST had no other statistically significantly associations with academic skills in all children (Table 2). Furthermore, sex modified many associations of MVPA, light PA, and ST with reading and arithmetic skills (Table 2).

In boys, higher levels of MVPA in Grade 1 were related to better reading fluency in Grades 1–3 and better reading comprehension and arithmetic skills in Grade 1 after adjustment for age and sensor wear time (Table 2). Higher levels of ST in Grade 1 were associated with poorer reading fluency in Grades 1–3 and poorer arithmetic skills in Grade 1 after adjustment for age and sensor wear time. Light PA in Grade 1 was related to better reading fluency in Grade 3. However, this association was no longer statistically significant after further adjustment for MVPA, ST, or body fat percentage (P > 0.100). Only the association of MVPA with reading fluency in Grade 2 remained statistically significant after mutual adjustment for MVPA and ST. Further adjustment for reading fluency in Grade 1 attenuated the associations of MVPA or ST in Grade 1 with reading fluency in Grade 2 (*P* > 0.150) but it had no effect on the relationships of MVPA or ST to reading fluency in Grade 3 (*P* < 0.030). Furthermore, the relationships of MVPA and ST in Grade 1 to reading and arithmetic skills in Grade 1 were no longer statistically significant after additional adjustment for body fat percentage or 50-m shuttle run test time (*P* > 0.065). The relationship between MVPA and arithmetic skills in Grade 1 was also attenuated after controlling for the wellbeing score (*P* = 0.092). Additional adjustments had no effects on these associations (data not shown).

In girls, lower levels of light PA and higher levels of ST in Grade 1 were related to better arithmetic skills in Grade 2 after adjustment for age and sensor wear time (Table 2). These associations were no longer statistically significant after mutual adjustment for light PA and ST (data not shown). The relationship of ST in Grade 1 to arithmetic skills in Grade 2 was no longer statistically significant after further controlling for body fat percentage. Additional adjustments had no effect on this association.

**Fig. 1:**
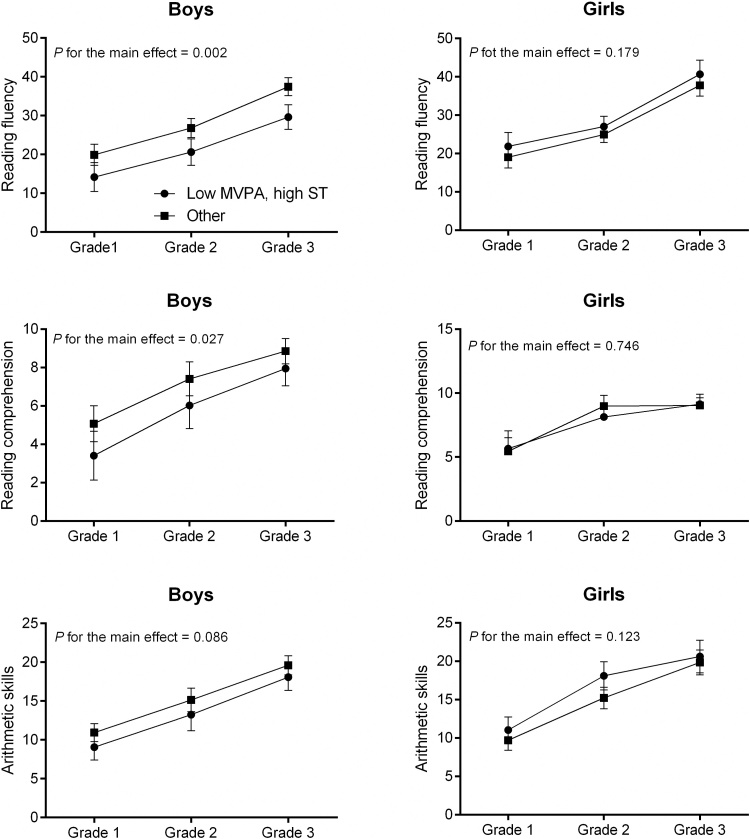
Differences in reading fluency, reading comprehension, and arithmetic skills in Grades 1–3 between 30 boys with low levels of MVPA and high levels of ST and other 56 boys and between 22 girls with low levels of MVPA and high levels of ST and other 37 girls adjusted for age and sensor wear time. The data are presented as estimated marginal means and their 95% confidence intervals.

Boys with a combination of lower levels of MVPA and higher levels of ST in Grade 1 had poorer reading fluency (mean difference across Grades 1–3 = −6.6, 95% CI for the difference = −10.6 to −2.6, *P* = 0.002) and reading comprehension (mean difference across Grades 1–3 = −1.3, 95% CI for the difference = −2.5 to −0.2, *P* = 0.027) than all other boys after adjustment for age and sensor wear time (Fig. 1). Boys with a combination of lower levels of MVPA and higher levels of ST also had poorer reading fluency in Grades 2–3 than all other boys after adjustment for age, sensor wear time, and reading fluency in Grade 1 (mean difference across Grades 2–3 = −3.0, 95% CI for the difference = −5.4 to −0.6, *P* = 0.014). In girls, no statistically significant combined associations of MVPA and ST in Grade 1 with reading and arithmetic skills in Grades 1–3 were found. Further adjustments had no effects on these differences (data not shown).

## 4 Discussion

In the present study among Finnish primary school children aged 6–8 years, we found that lower levels of MVPA, higher levels of ST, and particularly their combination, were related to poorer reading skills in boys. Furthermore, we observed that higher levels of MVPA and lower levels of ST were related to better reading skills in Grades 2 and 3, independent of reading skills in Grade 1 among boys. We found few associations of PA and ST with academic skill in girls. Lower levels of light PA and higher levels of ST were associated with better arithmetic skills but the relationship between ST and arithmetic skills weakened markedly after controlling for body fat percentage.

Previous investigations suggest either direct,^8^ non-significant,[5], [10], [17] or inverse^9^ relationships between objectively measured MVPA and academic achievement in children and adolescents. We found a direct association between MVPA and academic achievement in boys, while the relationship was statistically non-significant or inverse in girls. Our observation on the direct associations of MVPA with reading skills in boys is in line with the results of studies showing direct relationships of PA and cardiorespiratory fitness to academic achievement and working memory in boys but not in girls at 6–10 years of age.[11], [12] Nevertheless, some studies in adolescents suggest a direct association between PA and academic achievement in girls but not in boys.[13], [14] It has been hypothesized that these sex differences may be due to more advanced sexual maturation and higher plasma levels of circulating sex-hormones, or better psychosocial wellbeing among girls than boys.[12], [28] Another reason for these sex-dependent associations may be that the effects of PA on self-esteem or friendship networks, which may increase school connectedness and school adjustment and thereby improve academic achievement, are larger in boys than in girls 6–8 years of age.[29], [30] Furthermore, girls may receive more educational support from their parents than boys^31^ which may have an effect on the associations of PA and ST with academic achievement in children during early school years. Weak and inconsistent associations of PA and ST with academic achievement among girl may also be partly due to a smaller number of girls than boys in our study sample and therefore limited statistical power in the analyses. Furthermore, body fat percentage weakened the association of ST with arithmetic skills in girls. More research is warranted on the effects of these factors on the association between PA and academic achievement.

Biological mechanism by which higher levels of PA may improve academic achievement in boys include improved neuroelectric processing, increased hippocampal volumes and plasma neurotrophic factor concentrations, enhanced blood flow in the brain, and improved attention and working memory.[2], [3] Furthermore, some evidence suggests that high levels of ST that result in low energy expenditure may impair learning by decreasing plasma levels of brain-derived neurotrophic factor.^2^ PA and ST likely have similar effects on the brain in girls and in boys, but other factors, such as parental educational support, peer acceptance, teachers’ positive attitude for the student, and children’s motivation towards school are more important correlates of academic achievement than PA and ST among girls.[32], [33]

Higher levels of TV watching and lower levels of reading have been associated with poorer academic achievement in children.[4], [11] There are few studies on the associations of objectively measured ST with academic achievement in children. Some of these studies have shown a weak positive relationship and other studies no association between ST and academic achievement.[5], [16], [17] We found that higher levels of objectively measured ST were related to poorer reading fluency in boys independent of screen-based sedentary behavior and reading. However, these associations were weakened after controlling for MVPA, suggesting that boys with higher levels of ST also had lower levels of MVPA. Accordingly, boys with a combination of lower levels of MVPA and higher levels of ST had a 6.5 point lower score in reading fluency and a 1.3 point lower score in reading comprehension than other boys. Thus, our data suggest that a combination of low MVPA and high ST might be particularly harmful for the development of academic skills in boys and that increasing MVPA, reducing ST, or especially both of them may improve academic achievement.

We found that the associations of MVPA and ST with reading and arithmetic skills in Grade 1, but not in Grades 2–3, weakened after controlling for body fat content and motor performance. It is well characterized that PA, adiposity, and motor performance are interrelated.^34^ Motor performance has been directly related to academic skills in children, particularly in boys[18], [25] and adiposity has been inversely associated with academic achievement in children.^35^ Although our results suggest that more active boys had a lower body fat percentage, better motor performance, and better reading skills in Grade 1 than other boys, physically active lifestyle in Grade 1 may improve reading skills later regardless of the level of adiposity and motor performance. Furthermore, higher levels of MVPA in Grade 1 were associated with better reading fluency in Grade 3 and the combination of high levels of MVPA and low levels of ST was related to better reading fluency in Grades 2–3 independent of reading skills in Grade 1 and other confounding factors. Thus, in line with the findings by Booth et al.,^8^ our results suggest that boys with lower levels of MVPA and higher levels of ST in Grade 1 had smaller improvements in reading skills during the first school years than other boys. An explanation for our observations might be that higher levels of MVPA and lower levels of ST in Grade 1 are related to improved brain structures and functions, better cognitive functions, self-esteem, school connectedness, and school adjustment that may improve learning particularly in boys.[3], [28]

The strengths of the presents study are the objective measures of PA and ST, the assessment of reading and arithmetic skills using standardized tests, and the prospective study design. We also used a comprehensive battery of important covariates in the analyses. However, our data do not allow us to draw conclusions on causal relationships, because we assessed PA and ST only in Grade 1. We also had a relatively small sample size particularly for girls that decreased power to detect statistically significant associations. Nevertheless, the characteristics of the study sample were relatively comparable to the excluded children. Because of a small sample size, we were not able to investigate the combined associations of MVPA and ST with academic skills using four possible groups of MVPA and ST but compared children with lower levels of MVPA and higher levels of ST with all other children.

## 5 Conclusion

In conclusion, lower levels of MVPA, higher levels of ST, and especially their combination were related to poorer reading skills in Grades 1–3 among boys. We found weak and inconsistent associations of PA and ST with academic skills in girls. Thus, our results provide some evidence that promoting a physically more active lifestyle may benefit the development of reading skills in boys during the first school years. More research is needed to investigate whether the associations of PA and ST with academic skills are different in boys and girls.

## Practical implications

– Increasing daily physical activity and decreasing sedentary time may improve academic performance particularly in boys.
– A combination of low levels of physical activity and high levels of sedentary was strongly related to poor academic performance in boys.
– Physical activity or sedentary time had small if any association with academic performance in girls.

## Figures and Tables

**Table 1:** Basic characteristics.

	All	Boys	Girls	*P*
Background characteristics				
Age (years)	7.7 (0.4)	7.7 (0.4)	7.6 (0.3)	0.021
Prepubertal (%)	96.8	96.7	97.0	0.935
Body height (cm)	130 (5.5)	130.0 (5.8)	128.5 (5.0)	0.052
Body weight (kg)^†^	25.8 (6.1)	26.4 (5.8)	25.5 (6.0)	0.250
Body fat percentage^†^	18.1 (11.2)	14.8 (11.6)	19.9 (10.4)	<0.001
Maximal work load/lean body mass (W/kg)	3.6 (0.5)	3.7 (0.5)	3.4 (0.4)	<0.001
50-m shuttle run test time (s)	24.1 (2.2)	23.8 (2.1)	24.7 (2.1)	0.012

Parental education (%)				
Vocational school or less	20.0	23.9	14.7	0.105
Polytechic	38.1	31.5	47.1	
University degree	41.9	44.6	38.2	

Household income (%)				
≤30,000	20.3	21.1	19.1	0.616
>30,000–60,000	45.6	42.2	50.0	
>60,000	34.2	36.7	30.9	

Risk of reading disabilities	13.8	18.5	7.4	0.043

Physical activity and sedentary time				
Sensor wear time during weekdays (hours)^†^	98.6 (49.4)	92.6 (45.1)	113.6 (52.5)	0.223
Sensor wear time during weekend (hours)^†^	40.8 (24.1)	38.7 (25.4)	45.0 (19.8)	0.170
Sedentary time (min/d)^†^	226 (192)	226 (199)	223 (202)	0.694
Light physical activity (min/d)	501 (117)	489 (112)	517 (122)	0.140
Moderate-to-vigorous physical activity (min/d)^†^	95.8 (82.7)	108 (88.9)	77.7 (62.6)	<0.001
				
Academic achievement				
Grade 1				
Reading fluency (range 0–28)	18.7 (9.5)	17.8 (10.4)	19.9 (8.0)	0.147
Reading comprehension (range 0–12)^†^	5.0 (5.0)	4.0 (6.0)	5.0 (6.0)	0.134
Arithmetic skills (range 0–28)	10.3 (4.2)	10.3 (4.4)	10.2 (4.0)	0.856

Grade 2				
Reading fluency	24.8 (8.4)	24.3 (9.5)	25.5 (6.8)	0.337
Reading comprehension	8.0 (5.0)	7.0 (5.0)	9.0 (4.0)	0.003
Arithmetic skills	15.4 (5.1)	15.0 (5.5)	16.0 (4.4)	0.176

Grade 3				
Reading fluency	36.4 (9.0)	34.7 (9.3)	38.6 (8.3)	0.007
Reading comprehension	9.0 (2.0)	9.0 (3.0)	10.0 (2.0)	0.126
Arithmetic skills	19.7 (4.8)	19.5 (4.6)	20.0 (5.1)	0.508

Data are from the Student t-test or Mann–Whitney U test for continuous variables and chi-square test for categorical variables and are displayed as means (SD) for normally distributed variables, medians (IQR)^†^ for slightly skewed variables, or percentages (%). *P* values refer to statistical significance for differences between boys and girls.

**Table 2:** Associations of physical activity and sedentary time with academic achievement.

	Reading fluency				Reading comprehension				Arithmetic skills			
	All	Boys	Girls	P for interaction	All	Boys	Girls	P for interaction	All	Boys	Girls	P for interaction
	Grade 1											
Sedentary time (min/d)	−0.111	−0.247*	0.147	**0.016**	−0.097	−0.209	0.034	0.132	−0.089	−0.229*	0.149	0.063
Light physical activity (min/d)	0.028	0.138	−0.146	0.133	0.067	0.109	0.035	0.653	0.045	0.174	−0.166	0.109
Moderate-to-vigorous physical activity (min/d)	0.163	0.285**	−0.171	**0.011**	0.105	0.255*	−0.211	**0.007**	0.133	0.227*	−0.108	0.094

	Grade 2											
Sedentary time (min/d)	−0.129	−0.225*	0.028	0.119	−0.103	−0.118	−0.133	0.892	0.028	−0.152	0.379**	**0.009**
Light physical activity (min/d)	0.042	0.071	−0.001	0.715	0.076	0.033	0.191	0.438	−0.025	0.164	−0.385**	**0.006**
Moderate-to-vigorous physical activity (min/d)	0.192*	0.309**	−0.183	**0.008**	0.113	0.190	−0.056	0.148	0.014	0.067	−0.155	0.309

	Grade 3											
Sedentary time (min/d)	−0.146	−0.354**	0.158	**0.004**	−0.080	−0.134	0.066	0.324	−0.032	−0.155	0.163	0.111
Light physical activity (min/d)	0.035	0.227*	−0.249	**0.015**	0.078	0.120	−0.037	0.455	−0.002	0.129	−0.203	0.105
Moderate-to-vigorous physical activity (min/d)	0.275**	0.350**	0.100	0.238	0.130	0.156	0.001	0.500	0.086	0.134	−0.036	0.431

Data are standardized regression coefficient from multivariate linear regression analyses adjusted for age, sex, and sensor wear time. Moderate-to-vigorous physical activity was defined as activities exceeding the intensity of 4 metabolic equivalents (METs), light physical activity as activities that were performed at the intensity of 1.5–4 metabolic equivalents (METs), and sedentary time as activities that were performed below the intensity of 1.5 METs. There were 153 children (89 boys, 64 girls) in Grade 1, 149 children (87 boys, 62 girls) in Grade 2, and 145 children (86 boys, 59 girls) in Grade 3.

Statistically significant associations are bolded.

* *P* ≤ 0.05.

** *P* ≤ 0.01.
